# Magnetic and magnetotelluric data integration to determine the origin of Siwa Oasis Lakes, Western Desert, Egypt

**DOI:** 10.1038/s41598-025-20074-x

**Published:** 2025-09-24

**Authors:** Mohamed Aldeep, Mamdouh Soliman, Hany S. A. Mesbah, Wael R. Gaweish, Ahmed M. Ali, Naser Meqbel, Mohamed Abdel Zaher

**Affiliations:** 1 The National Research Institute of Astronomy and Geophysics (NRIAG), Helwan, Cairo, Egypt; 2 3D Consulting-GEO GmbH, Berlin, Germany; 3 The National Observatory of Brazil, Rio de Janeiro, RJ Brazil; 4 Department of Geoinformatics, Physical and Environmental Geography, University of Szeged, Egyetem U. 2-6., Szeged, 6722 Hungary

**Keywords:** Magnetotelluric, Magnetic, Groundwater, Inversion, Solid Earth sciences, Geophysics

## Abstract

Geological structures are the most critical parameter for understanding the distribution of groundwater, oil, and minerals. In Siwa Oasis and other oases with comparable geological settings in the northern Western Desert, Egypt, brackish groundwater seeps to the surface through fractures and permeable zones, sustaining phreatophytic vegetation and enabling human habitation in otherwise arid environments. The formation of these oases is closely tied to the subsurface structural and hydrogeological conditions. To determine the origin of these lakes, a geophysical investigation was conducted using magnetic and magnetotelluric (MT) data to identify whether the water leakage stems from deeper artesian aquifers or shallower marine-origin reserves. Magnetic data were used for the geological structural analysis and the depth estimation of basement rocks. The Magnetic Data Analysis includes constructing the Total Horizontal Gradient (THG), Tilt Derivative (TDR), and 2D Magnetic Modelling Constrained by borehole data to estimate basement depths. Magnetotelluric (MT) data analysis includes obtaining 3d resistivity models via inversion, and Cross-sections were extracted to trace fault zones and lithological contrasts. The Key Findings from this study are Surface Lineaments that are dominant in the Northeast (NE), North (N), and East–Northeast (ENE), with a minor East–West (E–W) trend. Shallow Trend Analysis (Tilt Derivative—TDR): Revealed structures in NE-SW, NW–SE, and E–W directions. Total Horizontal Gradient (THG) Map: Confirmed major structural trends in NE–SW, NW–SE, and E–W directions, with additional W–NW trends. The E–W trend correlates with most lakes in the study area. Depth estimation (2D magnetic modelling): Basement rock depths range between 3400 and 4600 m in the magnetic data coverage. The magnetotelluric (MT) model reveals a near-surface E-W direction with resistivity variations linked to lithology changes and groundwater presence.

**Supplementary Information:**

The online version contains supplementary material available at 10.1038/s41598-025-20074-x.

## Introduction

The Siwa Oasis region is a geological enigma, revealing a profound and intricate tectonic history that influences its stunning topography and essential groundwater systems. The area, now inside a stable intra-craton portion of the North African Plate, has a fault system that documents reactivation and deformation events from early Precambrian formations to subsequent Mesozoic and Cenozoic tectonic occurrences^1,2^. The oasis has historically preserved distinct Amazigh cultural traditions and archaeological sites such as the Amun Oracle Temple, which is connected to civilization by Route 10 through Egypt’s Western Desert. Geomorphologically, the 900 km^2^ oasis constitutes part of the Qattara Depression. It is bounded northward by a 200-m-high Miocene limestone escarpment and southward by calcareous dunes reaching 20–50 m. The basin extends 80 km west–east but narrows to just 30 km north–south.

**Fig. 1:**
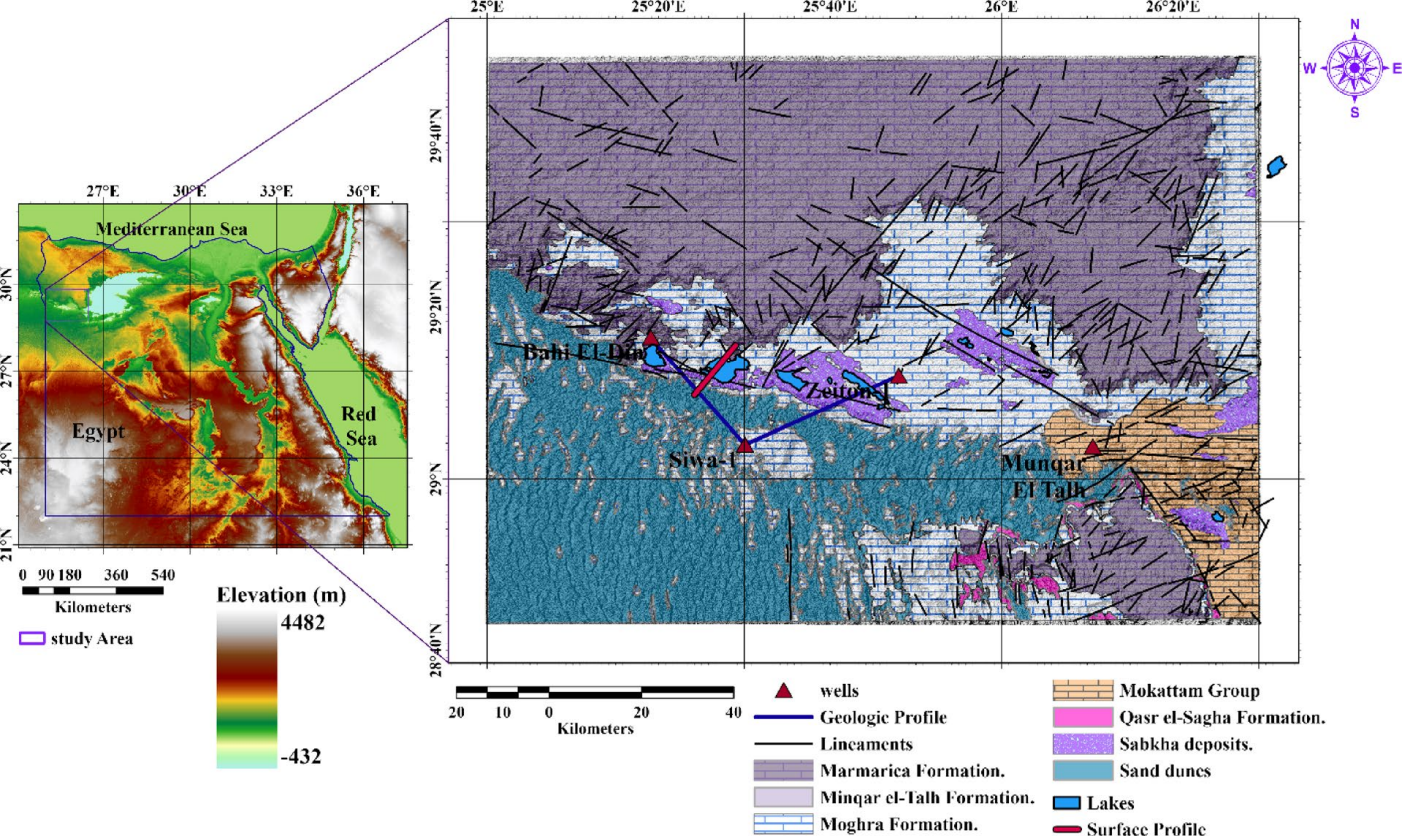
The left panel shows the location of the studied area, and the right panel shows the geological map of the study area^4^ (It was generated by ArcMap 10.8 software (https://www.esri.com/en-us/arcgis/products/arcgis-desktop/overview).

The leakage of brackish groundwater to form several lakes in the middle, western Egypt, and eastern Libya gives a source of water that causes the formation of the oasis. Figure 1 shows that the Siwa Oasis forms several seepage lakes. Geological structures—including faults, folds, fractures, and basement topography—play a pivotal role in controlling the distribution of groundwater, hydrocarbons, and mineral deposits. The map was generated by ArcMap version 10.8, where the left panel includes the location of the Study area with topography data as measured by the Shuttle Radar Topography Mission (SRTM)^3^, and the right panel is the geological map digitized from the Geological map of Siwah Quadrangle datasheet^4^.

### Study area

**Fig. 2:**
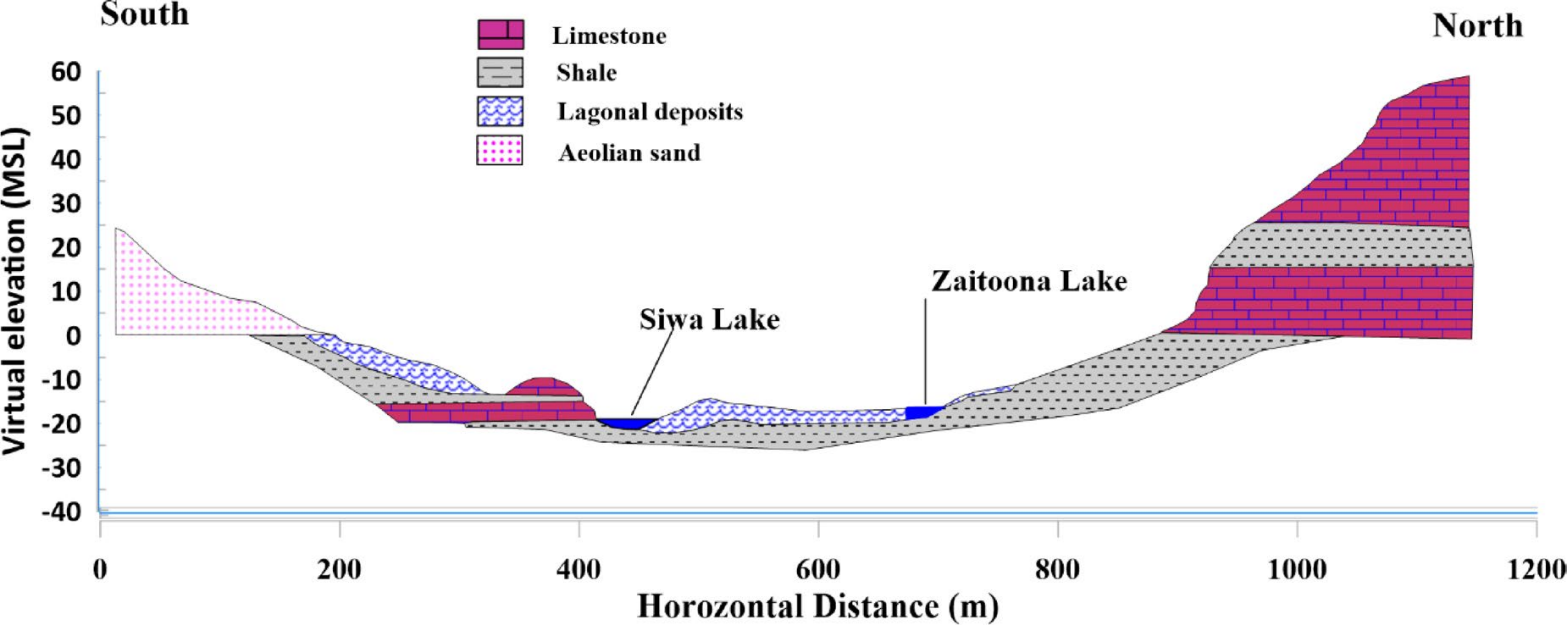
Schematic north–south surface profile (shown in Fig. 1) crossing Siwa Oasis, illustrating the contrast between the northern and southern banks, modified after^1^.

Siwa Oasis, situated in Egypt’s northwestern Matrouh Governorate, lies roughly 560 km southwest of Cairo and 300 km from Marsa Matrouh, close to the Libyan border (29.20°N, 25.52°E). This isolated desert region sits 18 m below sea level within the Sahara’s Great Sand Sea, forming a topographical depression flanked by extensive dunes and saline flats. Its hydrological system relies on ancient groundwater reserves from the Nubian Aquifer, which supplies multiple natural springs^5^. From a regional perspective, those lakes take the direction of East–West and show some patterns that may relate to structural features or unique sedimentary circumstances like ancient river courses. A north–south cross-section modified after^1^ is shown in Fig. 2, which shows the higher elevation of the northern plank, which is covered with Marmarica limestone.

The geological structures of Siwa Oasis are discussed by^6^ who described Siwa Oasis depression as an elongated, asymmetric, and irregular syncline. It has a west–east extension of more than 80 km and forms the middle part of the tremendous geomorphic features of the Jaghbub-Siwa-Qatlara depressions.^7^ used satellite gravity and magnetic data to build the structural framework of the Southern Faghur–Siwa Basin. They reported that the basin is dominated by N–S, NNW-SSW, NE-SW, and E-W trending faults. These structures influence fluid migration pathways, aquifer connectivity, and reservoir compartmentalization, making their analysis essential for resource exploration and sustainable management.

The hydrogeology in Siwa oasis descriped by^1^, where the groundwater system beneath Siwa Oasis consists of two principal aquifers: the lower Cretaceous Nubian sandstone and the middle Miocene fractured limestone, besides a third aquifer formed within the Quaternary sand and clay water-bearing beds. The former aquifer serves as the sole sustainable source of fresh groundwater in the Western Desert, with its thickness gradually diminishing from south to north, reaching approximately 200 m at Siwa oasis.

The geochemistry and groundwater quality were examined by^8^, revealing that the shallow aquifer in the Siwa Oasis has undergone significant evolution. It is notably enriched with marine residues, as evidenced by NaCl, MgCl_2_, MgSO_4_, CaSO_4_, and Ca (HCO_3_)_2_. These components result from the leaching and dissolution of both marine and terrestrial salts, contributing to the salinization of the carbonate aquifer in the region. ^9^conducted a geochemical and radiochemical analysis of the water in Siwa lakes. He found that the lake’s water is generated from springs and lakes related to enriched carbonate rocks, and the chemical fingerprint of the water is not associated with the deep Nubian aquifer.

The magnetic method was proven effective for geological structure detection by many authors, ^10,11^ and many authors have applied it to structural analysis from magnetic data-oriented for groundwater and minerals exploration^12–19^ On the other hand, magnetotelluric can be another powerful tool for detecting low resistivity layers, which may be an aquifer, in addition to structure analysis and interpretation. Many authors have used it for those applications^20–22^.

The following study presents an integrated geophysical approach to investigate Siwa lakes, combining magnetic surveys for mapping basement structures and estimating rock depths. Magnetotelluric (MT) Soundings—To image subsurface resistivity variations linked to lithology and fluid content. The main objective of this study is to determine whether the water leakage originates from Deep-seated artesian aquifers or Shallow marine-origin reserves, indicating relic seawater or sabkha-related brines. Also, to characterize structural trends controlling groundwater emergence, and estimate basement rock depth to assess basin geometry.

The available surface structure data were used to detect major surface lineaments. The magnetic method was applied for deeper structural analysis, which is why it covers a larger area than the AMT survey, and a magnetotelluric survey was conducted for 3D modeling of subsurface conductivity to evaluate factors controlling groundwater Movement in the study area.

## Geologic settings

**Fig. 3:**
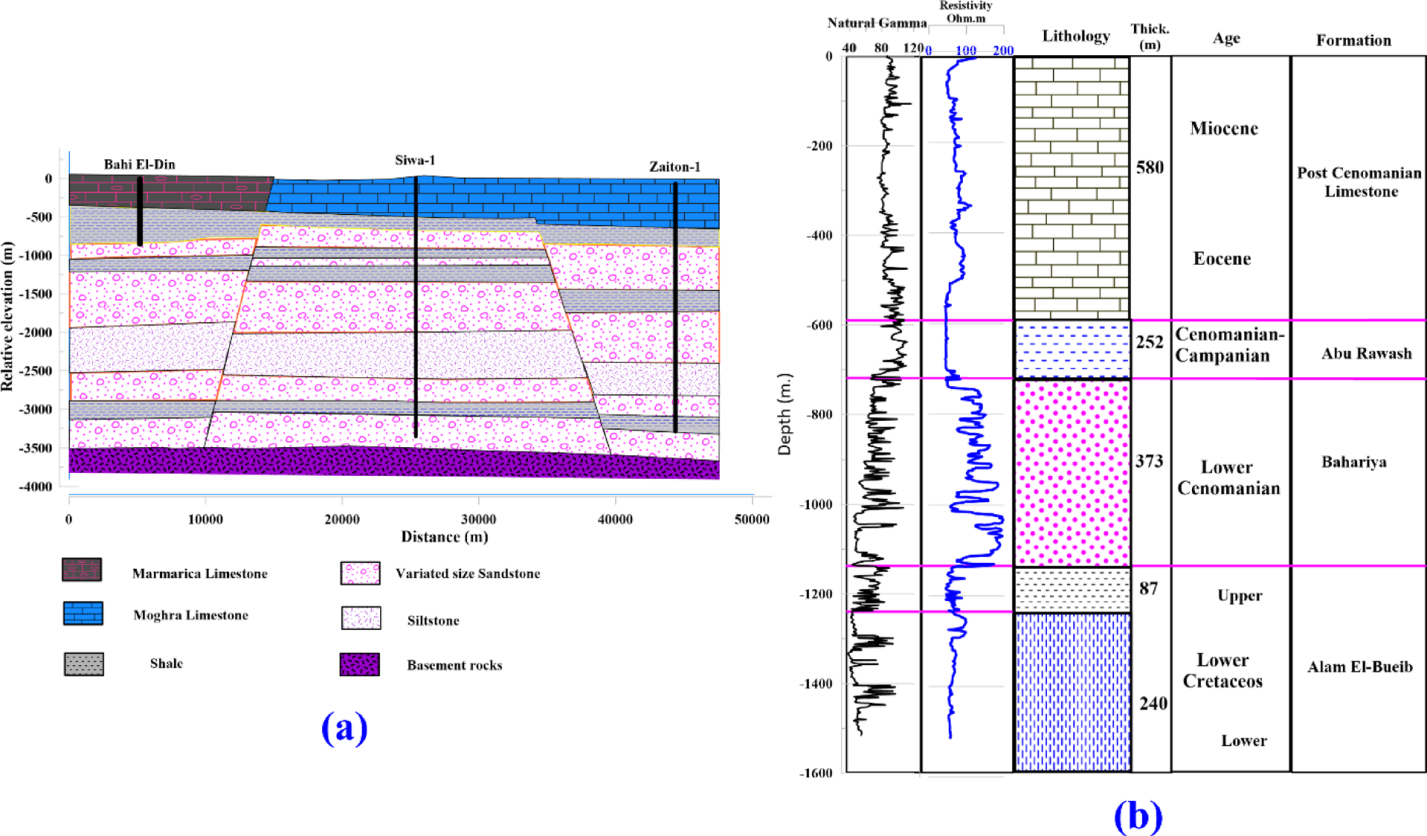
(**a)** geological cross-section (modified after^1^), (**b)** interpretation of well-logging data of well Munqar El-Talh (Modified after^2^^)^ in the studied area.

The basement lies 3 to 3.5 km below the ground surface in the Siwa area. This sequence is of Paleozoic, Mesozoic, and Tertiary age, and its sandstone facies are generally known as “Nubian Sandstone”. The lithostratigraphic units in Siwa are described in brief herein (starting from top-downward): a geologic cross-section generated from well-logging data and modified after^2^ shown in Fig. 3.

Quaternary sediments: In the Siwa depression, recent alluvium and aeolian deposits may be seen as dunes that border the depression to the south and as a thin layer resting on Tertiary rocks. In many depression areas, the soils are thinner and do not surpass 0.5 to 2 m in thickness. Most Quaternary deposits have a shallow water table, frequently with a capillary zone (nodose water) between the ground surface and the water table. Salt-crust deposits called “Sabkha” may be found in many low-lying locations. In addition to impeding movement, these Sabkha make agricultural development and reclamation challenging and expensive.

The Marmarica Formation represents the rocks of the Middle Miocene in the Siwa region. It is made up of shale, dolomite, and limestone. The stones of the whole northern scarp, including Gebel El Dakrour, Murtazaq (south of the Abu Shrouf region), Gebel El Mawta (3 km north of Siwa city), and Gebel Khamisah (west of Siwa Lake), are composed of the hard limestone of the Marmarica Formation. The scarp north of Siwa, whose surface section is 78 m thick (base unexposed), is the type location. The Moghra Formation, which grades laterally to the north and west into the maritime Mamura Formation, is a representative sample of lower Miocene rocks^23^. The traditional 230-m surface section in the east (Moghra Depression) comprises calcareous shale, siltstone, and sandstone.

In contrast, the western (Siwa region) portion comprises alternating sandstone, shale, and marl stages. There are a lot of gypsiferous shales. The strata of marl and limestone alternate as the formation ends upward. The uncovered portion of the Siwa Oasis is around 75 m thick.

In the southern portions of the Siwa-Qattara hollows and south of the eastern portion of the Siwa-Minqar El-Talh Road (70 km east-southeast of Siwa city), Eocene rocks are visible outside the Siwa depression, according to^24^. The Middle Eocene is when the carbonate rocks of the Apollonia Formation were deposited in the study region. Shale and marl strata are intercalated with limestone-based middle and upper Eocene rocks that are around 350 m thick below the surface. Evaporite streaks can occasionally be seen. Because of carbonate rocks and gypsum dissolution, the Eocene carbonates are characterized by karstification.

The Paleocene section is developed into chalk facies at the base and shale facies at the top. The shale section is interbedded with hard limestone beds and has a thickness of about 150 m. Together with the Upper Cretaceous shale beds, it acts as a confining layer above the Nubian sandstone aquifer system.

The oldest sediments belong to the Upper Cretaceous, which belongs to the Mesozoic Era and comprises shale, carbonate, and sandstone beds. The Upper Cretaceous (Cenomanian) sandstone and shale interbeds in the Siwa area are represented by the Bahariya Formation, which corresponds to the uppermost part of the Nubian sandstone aquifer.

### Structures

We can classify structure trends in the Siwa Oasis area into surface, shallow, and deeper structural trends. The nature of the Siwa lakes is more related to the near-surface structural trends^25^ detected. Four major vertical fracture zones were detected, corresponding to two conjugate sets of strike-slip faults that governed the surface and subsurface environments of the lakes in the Siwa region. These correlate well with the regional tectonics Landsat-7 ETM+/SRTM DEM-derived lineaments, the four major vertical to near vertical fracture zones detected are trending NW–SE, WNW–ES, NNE–, and NE–SW, and comprise two nearly perpendicular conjugate sets of faults.

## Methodology

The utilization of geophysical data accomplished the objectives of this research. The study of magnetic and magnetotelluric data included, in addition to the geological and geochemical work that had been done previously, a wide range of techniques, ranging from the straightforward digitization of geological cross-section and well-logging data to the inverse modeling of magnetic and magnetotelluric data.

### Magnetic data

**Fig. 4:**
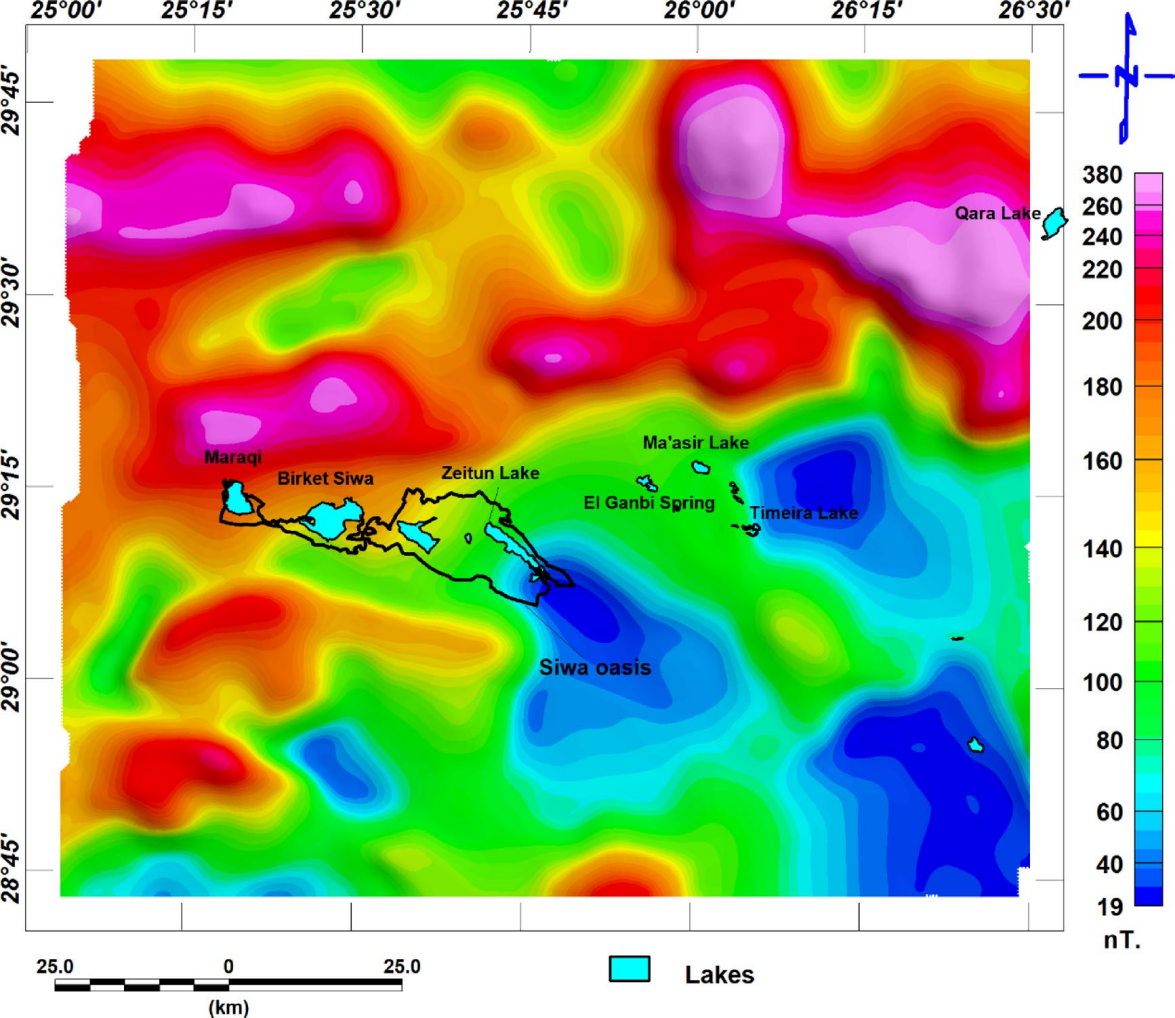
(**a)** The total magnetic intensity map with the brackish groundwater seepage lakes.

**Fig. 5:**
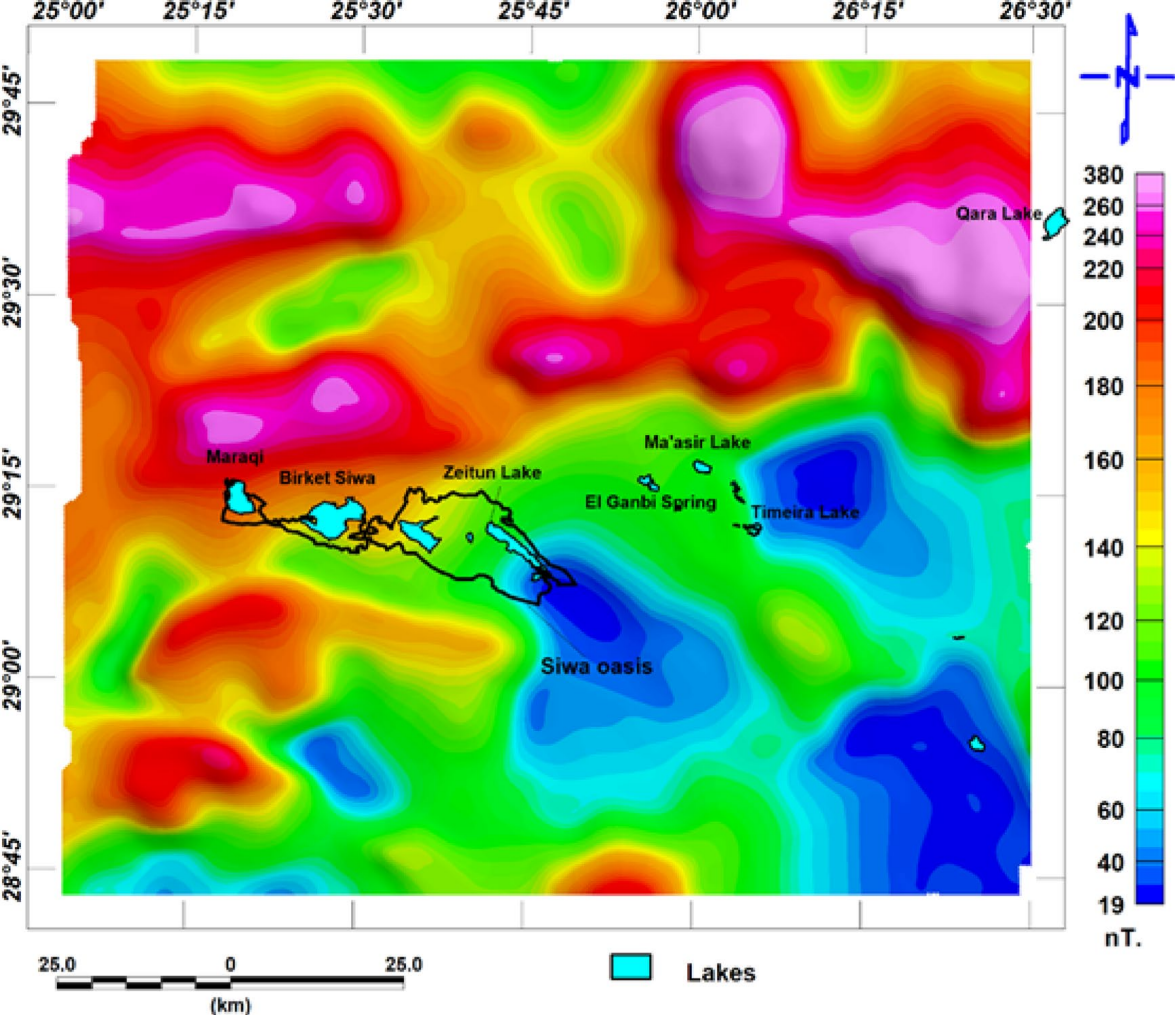
(**a**) the reduced to-pole map with the brackish groundwater seepage lakes.

This data was plotted as reduced to the magnetic pole map (RTP) and prepared by^26^ Matruh, and Baharyia during 1980. The data was collected with a terrain clearance of about 305 meters. It was filtered with an upward continuation of about 183 meters for smoothing and high-frequency noise. The International Geomagnetic Reference Field (IGRF) parameters used for earths main magnetic field removal where: H the magnetic field intensity equal to 41765 nanoteslas (nT), the inclination which refer to refers to the angle that the Earth’s magnetic field makes with the horizontal plane found to be equal to 40.5°, and declination 2.0° which present deviation of magnetic field direction with the true north. the total magnetic intensity is shown in Fig. 4. The magnetic data was processed and filtered using the software^27^. The RTP map is shown in Fig. 5.

A significant relationship exists between magnetic anomaly trends’ direction, pattern, and intensity was descriped by^10^ discussed the significance of the anomaly peaks affecting the basement rocks as follows: A sharp peak with a slight standard deviation may indicate a trend caused by fracturing of the uniform medium in response to the stress of constant direction; A broad peak with a significant standard deviation may be expected to be formed by successive renewals of shafting stress direction; A peak with moderate asymmetry may be formed by larger scale and more minor stresses in a different direction.

In this study, we applied some mathematical filters, such as the total horizontal gradient filter and the tilt derivative, in addition to the reduced-to-pole map, for the subsurface structural interpretation. These filters enhance linear features in the magnetic data by calculating the magnetic fields. The total horizontal gradient (THG) effectively identifies the edges of geological bodies and is widely used in mineral exploration and geological mapping.

The Tilt derivative (TDR)^28,29^ enhances the detection of shallow basement structures and mineral exploration targets. This filter improves the delineation of geological contacts and subtle features, making it particularly effective for targeting mineralized zones and structural boundaries. The TDR angle varies from -90 to 90 degrees, regardless of the amplitude of the magnetic data. It is calculated using the vertical derivative’s ratio to the magnetic field’s total horizontal derivative. The TDR was described and developed by^30–32^**.** The total horizontal derivative of the tilt derivative THDR or the horizontal gradient is described by^33^ and is defined as follows:

1 $$THDR = \sqrt {\left( {\frac{\partial T}{{\partial x}}} \right)^{2} + \left( {\frac{\partial T}{{\partial y}}} \right)^{2} }$$

The second derivative of $T$ the total magnetic field intensity in x and y directions, and finally, the tilt derivative TDR given from the following equations

2 $$TDR = \tan^{ - 1} \left( {\frac{dT/dz}{{\sqrt {\left( {dT/dx} \right)^{2} + \left( {dT/dy} \right)^{2} } }}} \right)$$

where $T$ is the total magnetic intensity derivative in the $x,y,z$ direction.

The analytic signal map was another technique related to the shape of the magnetic anomalies without the bias related to the inclination and declination of the magnetic field. The analytic signal can be used for structural analysis and depth estimation. The three-dimensional analytic signal was constructed using the following equation.

3 $$A\left( {x,y} \right) = \sqrt { \left( {\frac{\partial T}{{\partial x}}} \right)^{2} + \left( {\frac{\partial T}{{\partial y}}} \right)^{2} + \left( {\frac{\partial T}{{\partial z}}} \right)^{2} }$$

Geophysical techniques such as 2D magnetic modelling are used to deduce subsurface geological formations and analyze magnetic anomalies using the GM-system program. The program uses polygonal cross-Sects. (2D/2.5D) or prisms (3D). The 2.5D calculation assumes the body has a finite strike length. The algorithm calculates the total magnetic field anomaly based on the dipole nature of magnetic sources. It requires defining the Earth’s magnetic field parameters (Intensity, Inclination, Declination) and the magnetization of the source (direction and intensity).^34^ The generated model helps analyze subsurface structures, including dykes, ore deposits, and faults, for the purpose of conducting geological research. The mathematical foundation that Talwani’s theory provides allows for simulating the magnetic effect of two-dimensional structures with elaborate geometries. According to the theory, polygons can approximate the representation of geological bodies, which can then be used to compute the magnetic effect at the surface.

### Audio magnetotelluric data

Audio frequency magnetotelluric (AMT) is a geophysical exploration technique that examines subsurface resistivity changes by measuring natural electromagnetic fields across a broad frequency spectrum, often ranging from 0.1 to 20,000 Hz. The source of magnetotelluric is the electromagnetic signals that come from lightning strikes, changes in the ionosphere, and other natural sources to look into the structure of the Earth’s electrical conductivity below the surface. AMT is commonly used in mineral exploration, geothermal studies, groundwater investigations, and geological mapping. While the technique offers high resolution and is cost-effective, it requires careful data acquisition and processing to mitigate the effects of noise and ensure accurate interpretation of subsurface structures. In this method, a continuous measurement of the induced variation in a magnetic and electric field is recorded, and the measured time series is then subjected to frequency-domain transformation using Fast Fourier Transformation (FFT). The measurements were conducted using the Geode EM3D by Geometrics.

The area is characterized by sabkha deposits and lakes, which cover most of the region, making it difficult to access. The current study conducted four AMT profiles with 5, 6, and 10 km of spacing, and station separations ranged from 2.2 to 10 km. The profiles were oriented northward with deviations up to 10° to the east. The sampling rate varied according to the frequency band, covering a range of 40 to 93 Hz. Quality control includes removing noisy data that could lead to erroneous results. Specifically, when the time series is measured in segments (packs), noisy segments are removed before the Fast Fourier Transform (FFT) step. The Fast Fourier Transform (FFT) for the timeseries data is computed using MTPro software developed by Geometrics. The data was processed using the remote reference algorithm of^33^ for remote referencing, which reduces local noise by cross-referencing with a remote magnetic and electric field station. Additionally, it interpolates or weights FFT outputs within AMT dead bands (1–5 kHz), where natural signals exhibit weakness. After extracting the frequency bands, the apparent resistivity can be calculated using Eq. (5).

Magnetotelluric was first described by^36,37^. The theory behind MT measurements can be derived from Maxwell equations under the quasi-static approximation; the most important concepts can be summarized as follows: from the concept of skin depth, the depth where the strength of the field is reduced to e − ^1^ is known as the skin depth

4 $$\sigma \approx 503\sqrt {T\rho_{a} }$$

where $T$ is the period, $\rho_{a}$ Is the apparent resistivity at that period.

The amplitude of each period is used to calculate the impedance (Z) as follows after^38^

5 $$\rho_{a} = \left( {\omega \mu } \right)^{ - 1} \left| Z \right|^{2} = \left( {\omega \mu } \right)^{ - 1} \left[ {Z_{r}^{2} + Z_{i}^{2} } \right]$$

where $\omega$ is the angular frequency, $\mu$ is the magnetic permeability of a vacuum, $r, and i$ is the real and imaginary parts of the impedance.

The phase tensor is a mathematical construct derived from the MT impedance tensor. It provides information about the phase relationships between electric and magnetic fields, independent of galvanic distortion. Thekew of the phase tensor is a scalar parameter that quantifies the departure from lower-dimensional (1D or 2D) structures toward 3D complexity. The phase tensor skew angle (β) quantifies the asymmetry of the MT phase tensor (Φ), immune to galvanic distortion. It is defined after^39^ as follows:

6 $$\lambda = \frac{{\phi_{max} - \phi_{min} }}{{\phi_{max} + \phi_{min} }}$$

7 $$\beta = \frac{1}{2}arctan\left( {\frac{{\phi_{xy} - \phi_{yx} }}{{\phi_{xx} + \phi_{yy} }}} \right)$$

where $\lambda$ is the ellipticity, $\beta$ is the skew angle, and $\phi$ is the phase tensor, The interpretation of $\beta$ is as follows: if $\beta$ is equal to zero, it detects a 1D and 2D structure if. β > 3° that detect a 3D structure and strong anisotropy.

The inversion routine used in this study is the ModEM software developed by^38,40^ a tool designed for three-dimensional magnetotelluric (MT) modelling and inversion. This routine employs a nonlinear conjugate gradient (NLCG) inversion scheme that minimizes the data misfit and model roughness, penalizing deviations from a starting model. The computations for the inversion were carried out using parallel processing on a high-performance computing cluster, enabling efficient handling of the extensive data involved in the 3D inversion process.

The Nonlinear Conjugate Gradient (NLCG) after^41^**,** is an advanced optimization algorithm used to solve nonlinear minimization problems, particularly inverse problems such as those encountered in geophysical modelling, including magnetotelluric (MT) inversion. in this method unlike Newton-based methods (e.g., Gauss–Newton, Levenberg–Marquardt), NLCG does not require computing or storing the Hessian matrix, making it memory-efficient for high-dimensional problems, as all geophysical inversion In MT inversion, the goal is to minimize the misfit between observed and modeled data a simple mathematical description is given in the following equation after^38^:

8 $${\Phi }\left( m \right) = \parallel F\left( m \right)\parallel^{2} + \lambda R\left( m \right)$$

where: $m$ are the Model parameters (e.g., resistivity distribution), **F**(**m**): Forward modeling operator, R(**m**): Regularization term (e.g., Tikhonov, smoothness constraints), and $\lambda$ is the regularization parameter. Before the inversion processes, the data were checked for the calculated frequency-dependent coherency between electric and magnetic field components(coherency), where data with coherency below 0.7 were rejected.

## Results and dissection

### Structures control the area

The surface structural lineament is traced from the geological map of the Siwa area datasheet (NH 35 SW SIWA) constructed by^4^; the traced surface lineaments are shown in Fig. 5a. We can notice that areas covered with dunes do not have observable surface faults. Figure 5b. Shows the rose diagram of the dominant structures in the area. The northeast-southwest is the major trend, with second-order Northeast, north-northeast, and East Northeast, and the east–west direction exists in the area in a lower order. This direction is related to most of the lakes within the studied area.

**Fig. 6:**
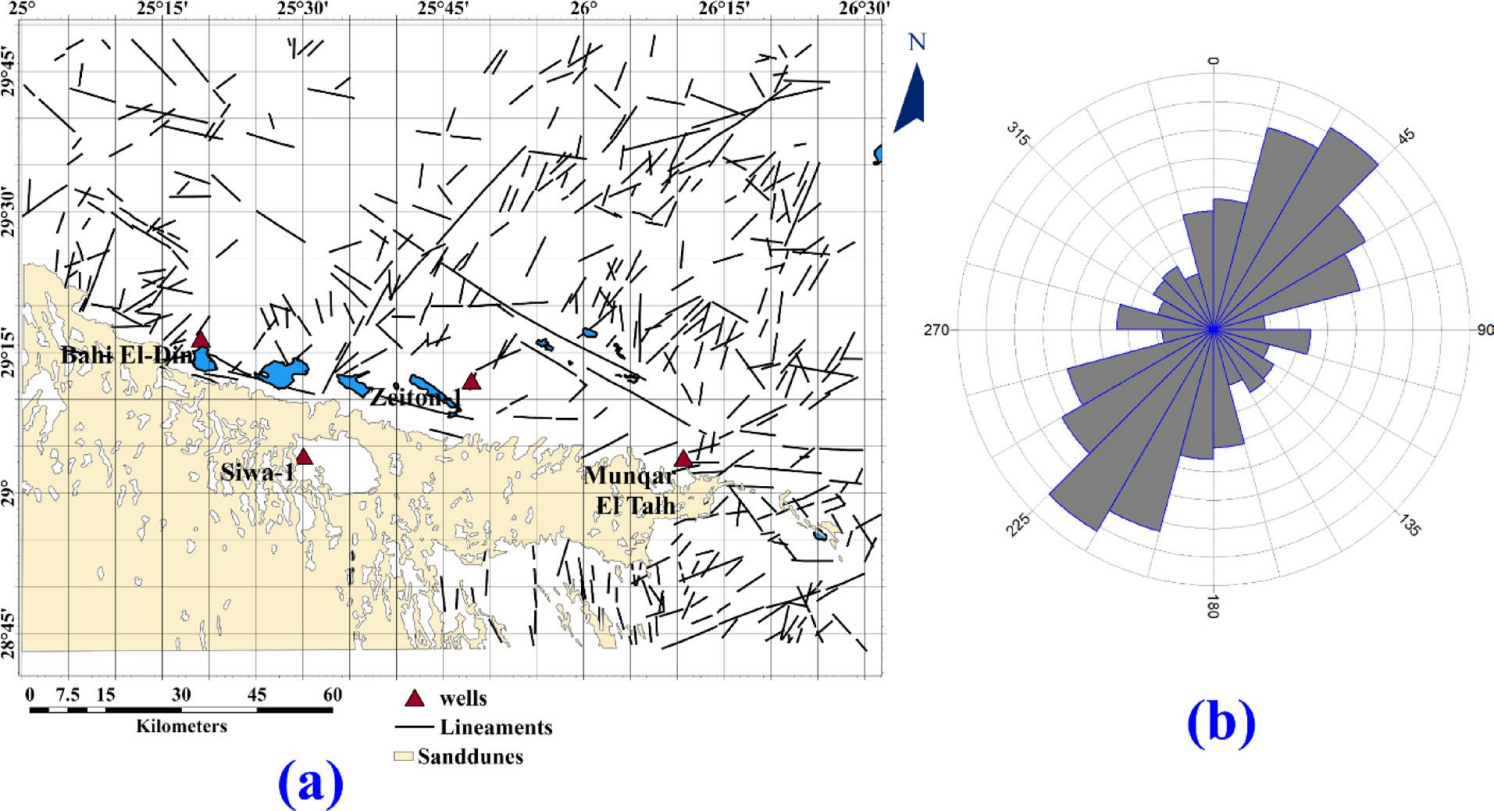
(**a**) Surface lineaments, as traced from^4^ as we see areas covered with dunes, missed outcrops for lineaments, and (**b**) The rose diagram shows the structure that dominated the area.

**Fig. 7:**
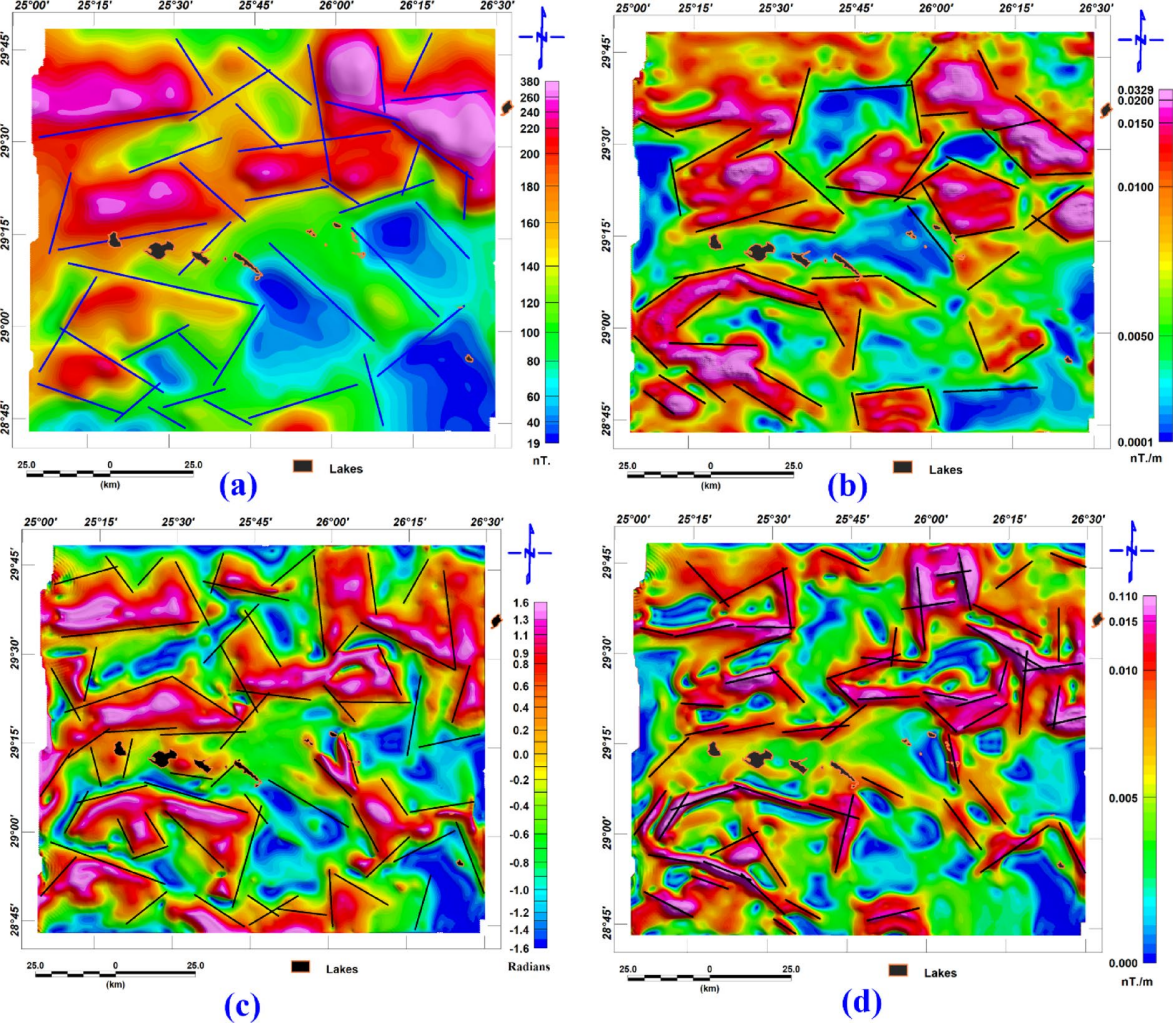
(**a**) the RTP map with the detected lineaments, (**b)** analytic signal map with lineaments, (**c)** Tilt derivative with detected lineaments, and d. total horizontal gradient with detected lineaments.

**Fig. 8:**
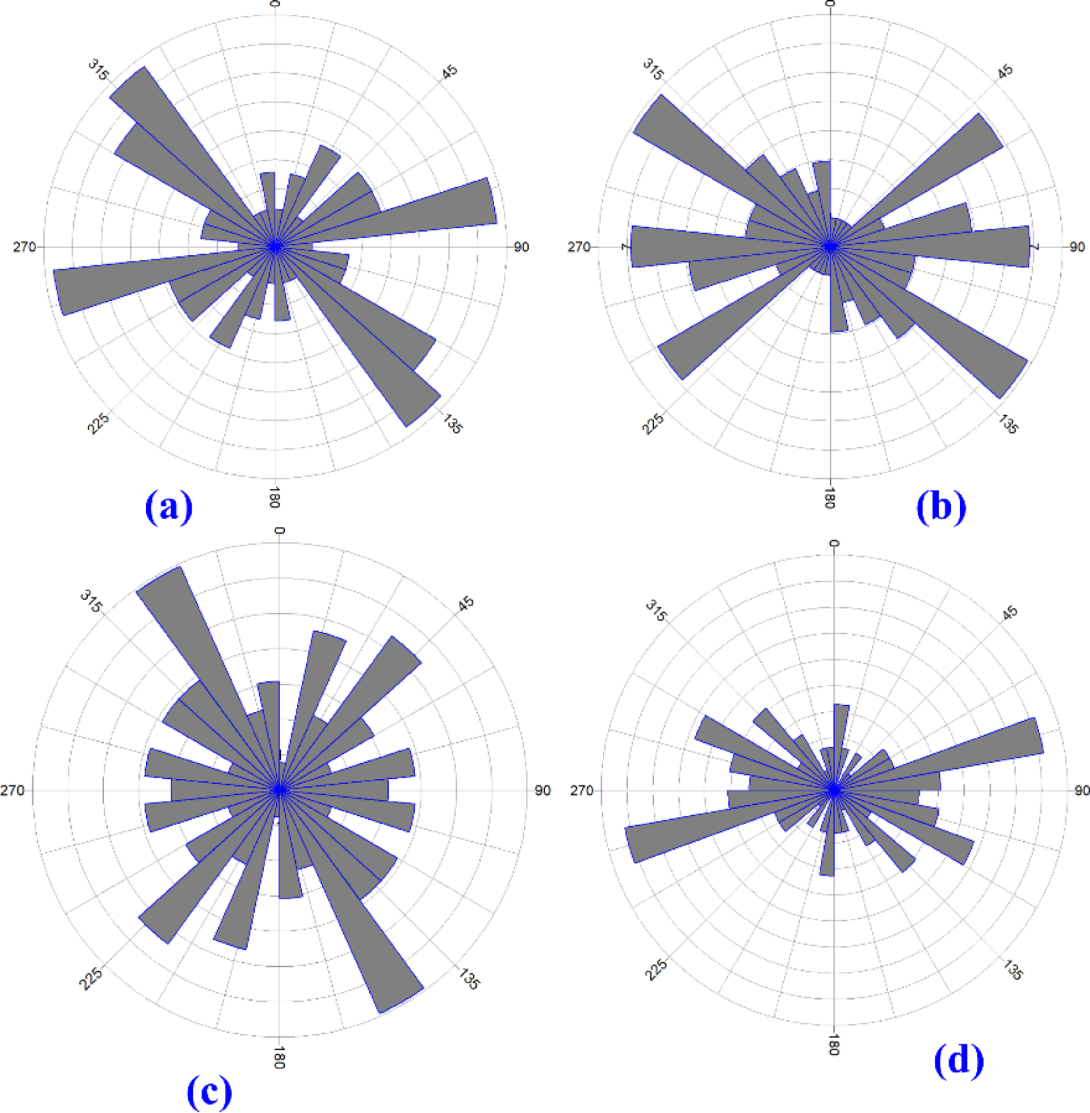
(**a**) rose diagram of the RTP, (**b)** rose diagram of the analytic signal map, (**c**) rose diagram of the tilt derivative (TDR), and d. rose diagram of the total horizontal gradient (THG).

Deeper Subsurface structural lineaments can be inferred from the lineaments traced from the RTP map and the analytic signal. Both maps are related to a prominent magnetic anomaly in the studied area, representing basement rocks, and are shown in Fig. 6a and b. respectively. The structure direction dominated in this area can be inferred from the rose diagram shown in Fig. (7a and b), respectively. The main structure trends are the northwest-southeast and the east–west direction (Fig. 8).

Shallow subsurface trends can be inferred from the lineaments traced from the tilt derivative and total horizontal gradient, which are shown in Fig. 6c and d, respectively; the filters related to shallower structures, and the rose diagram summarizing the structure trends are shown in Fig. 7c and d. The main structure trends from the TDR map are Northeast-southwest, northwest-southeast, and east–west direction; the main structure trends from the THG map are northeast-southwest, northwest-southeast, east–west direction, and west-northwest. The relation between shallow structures detected by magnetic data and a main imagery trend coinciding with the east–west direction is apparent. While some lakes are oriented to a northwest-southeast direction (Fig. 8).

### Depth estimation

**Fig. 9:**
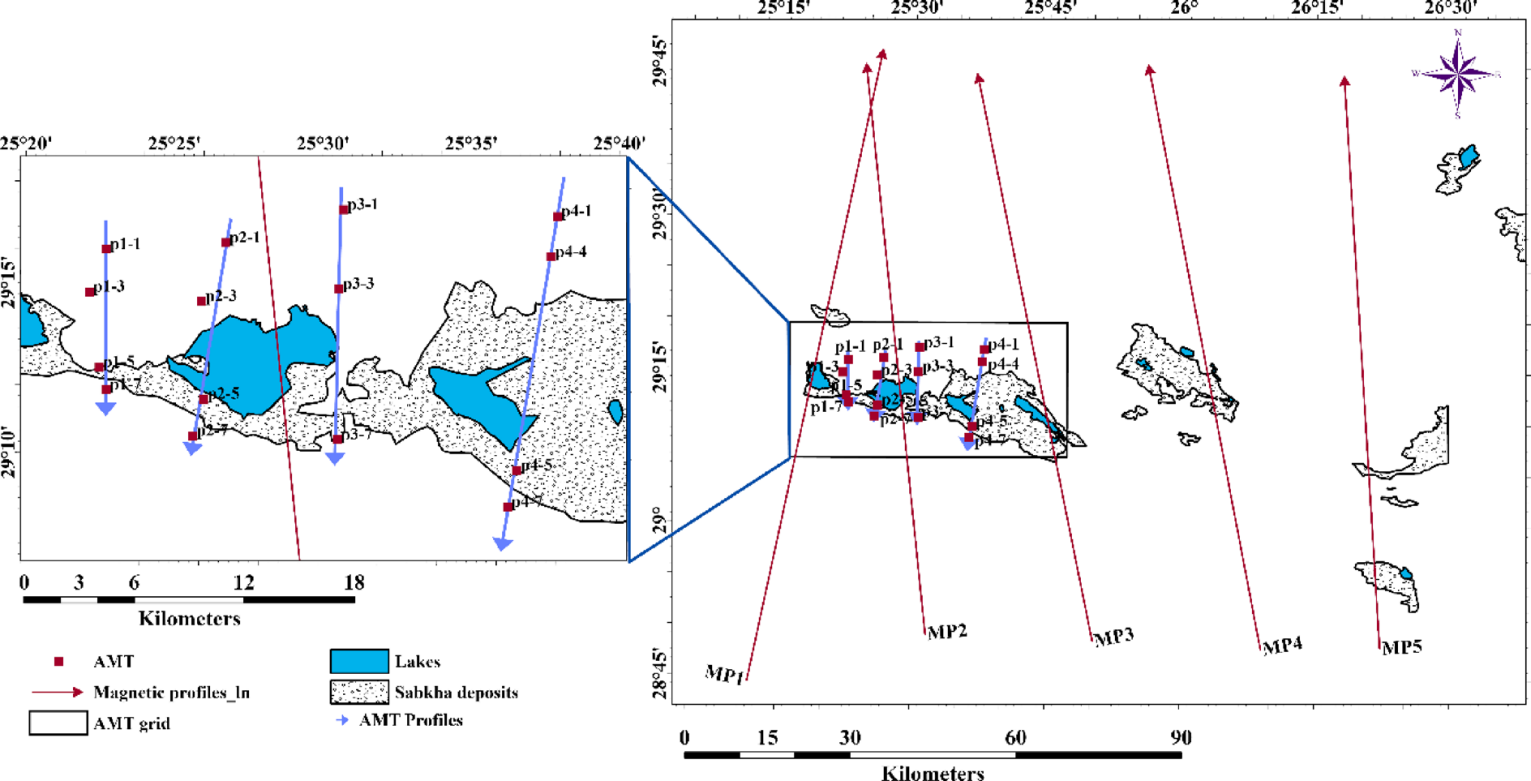
The left panel shows the AMT soundings distribution and profiles extracted from the 3D resistivity model in a blue color, with the arrowhead referring to the end of the cross-section, and the right panel shows the inverted Magnetic profiles.

**Fig. 10:**
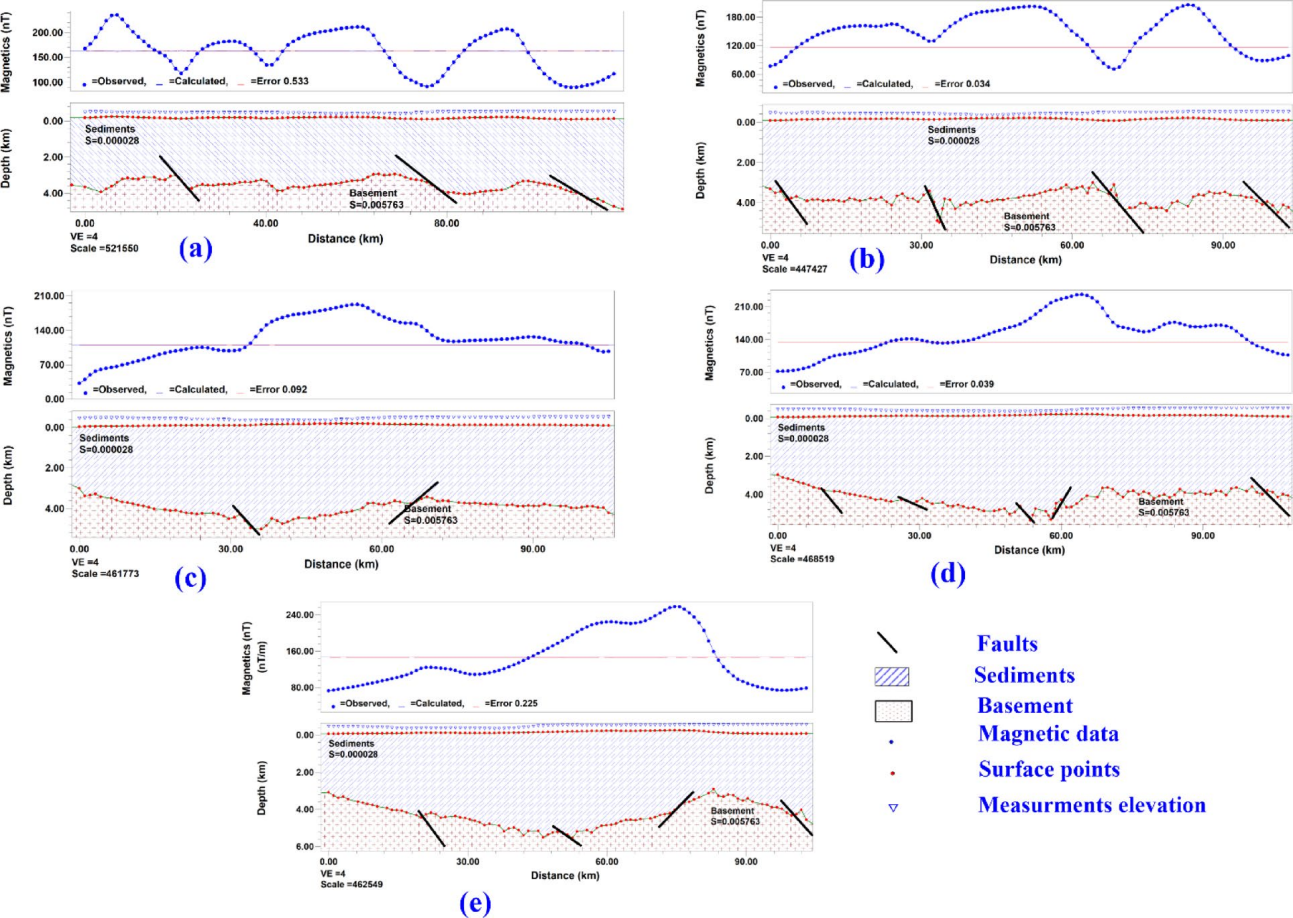
Inverted magnetic models of profiles presented in Fig. 8a–e are the inverted profiles of MP1 to MP5, respectively.

The inversion models for five magnetic data profiles oriented from south to north as shown in Fig. 9 as the arrow direction pointing, the models are shown in Fig. 10(a–e) for profiles MP1 to Mp5 respectively, all the inverted model constructed assuming 2-layer earth model where the magnetic susceptibility of the sedimentary cover is equal to 0.000028 CGS units. In contrast, the basement rocks representing the magnetic source assigned magnetic susceptibility 0.0057 CGS units, the basement topography surface fitted by Gm-system program, depth to basement varies from 3400 to 4600 m, and some faults are interpreted as shown on the models.

### Magnetotelluric Resistivity model

**Fig. 11:**
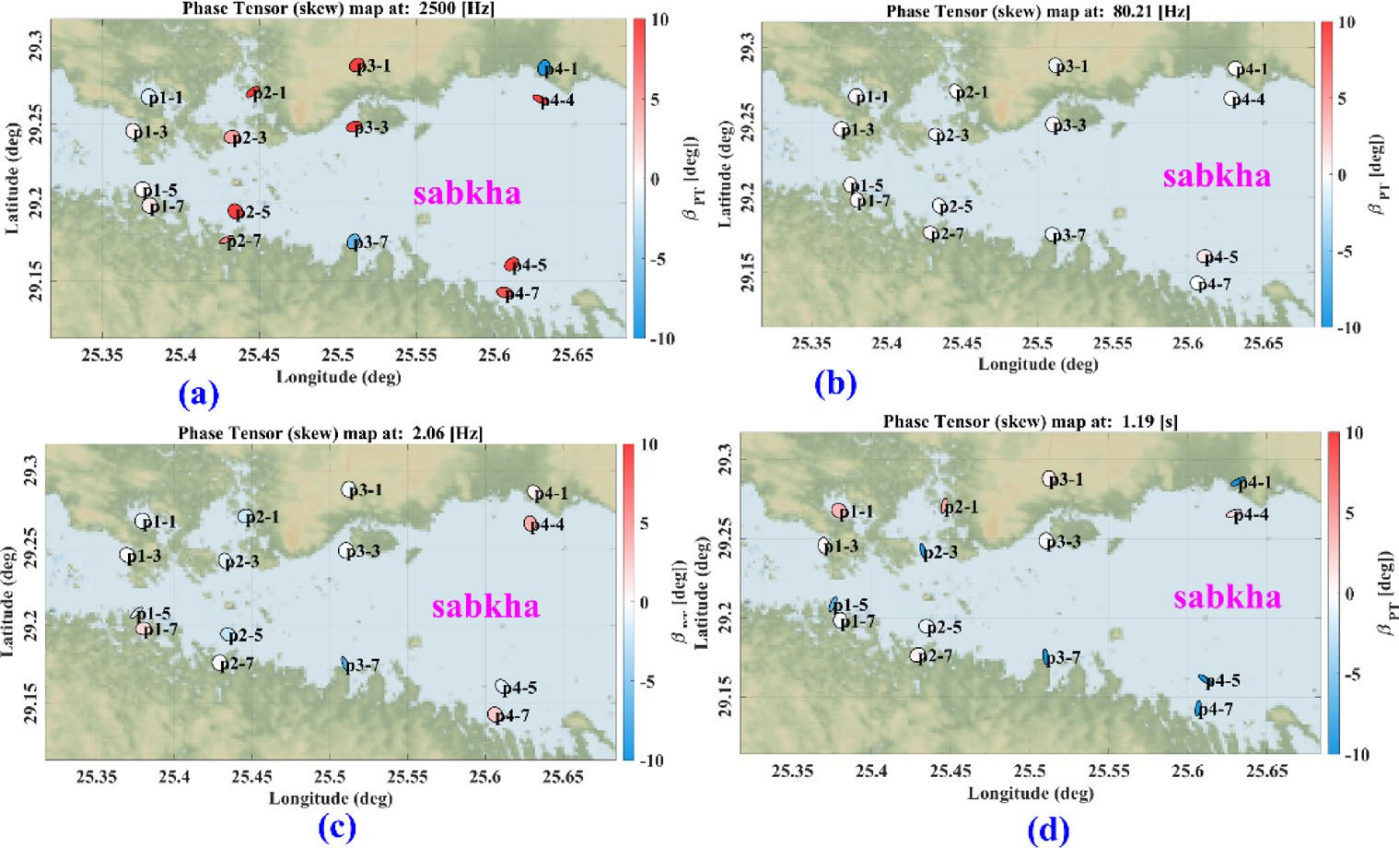
Shows the skew angle variation across selected frequencies.

**Fig. 12:**
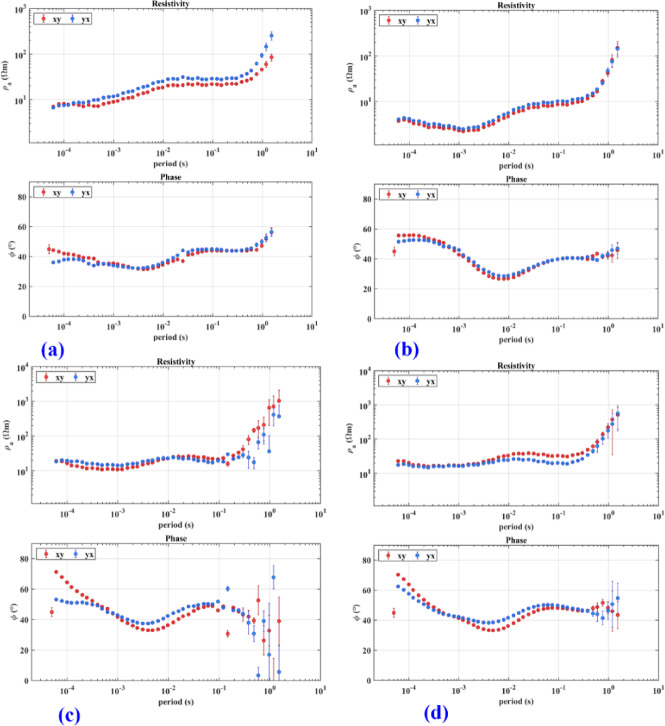
Apparent resistivity and phase measured at the profile (1), (**a**–**d**), are the AMT soundings arranged from north to south.

**Fig. 13:**
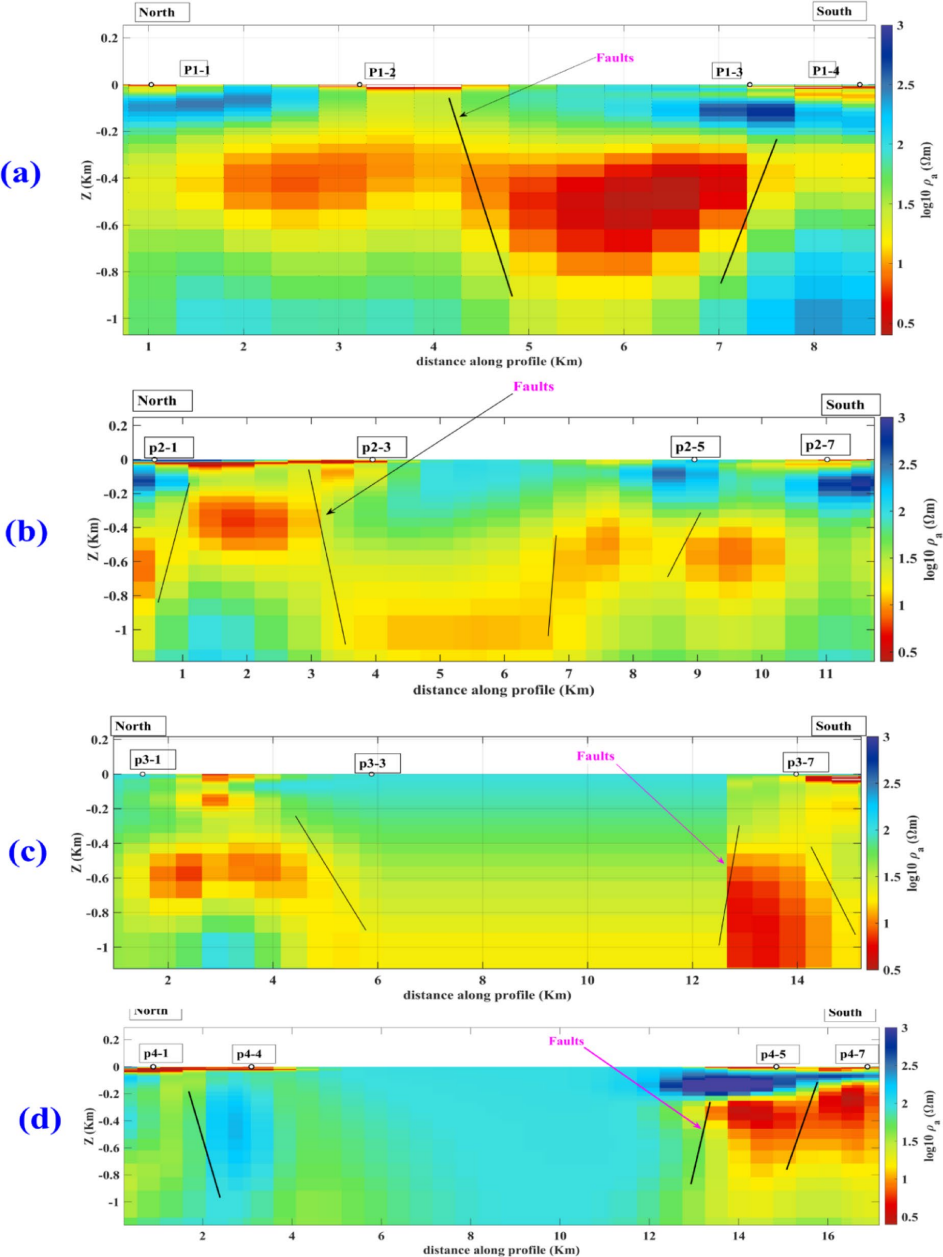
(**a**–**d**) are the geoelectric profiles p1, p2, p3, and p4 extracted from the 3D inverted MT model.

**Fig. 14:**
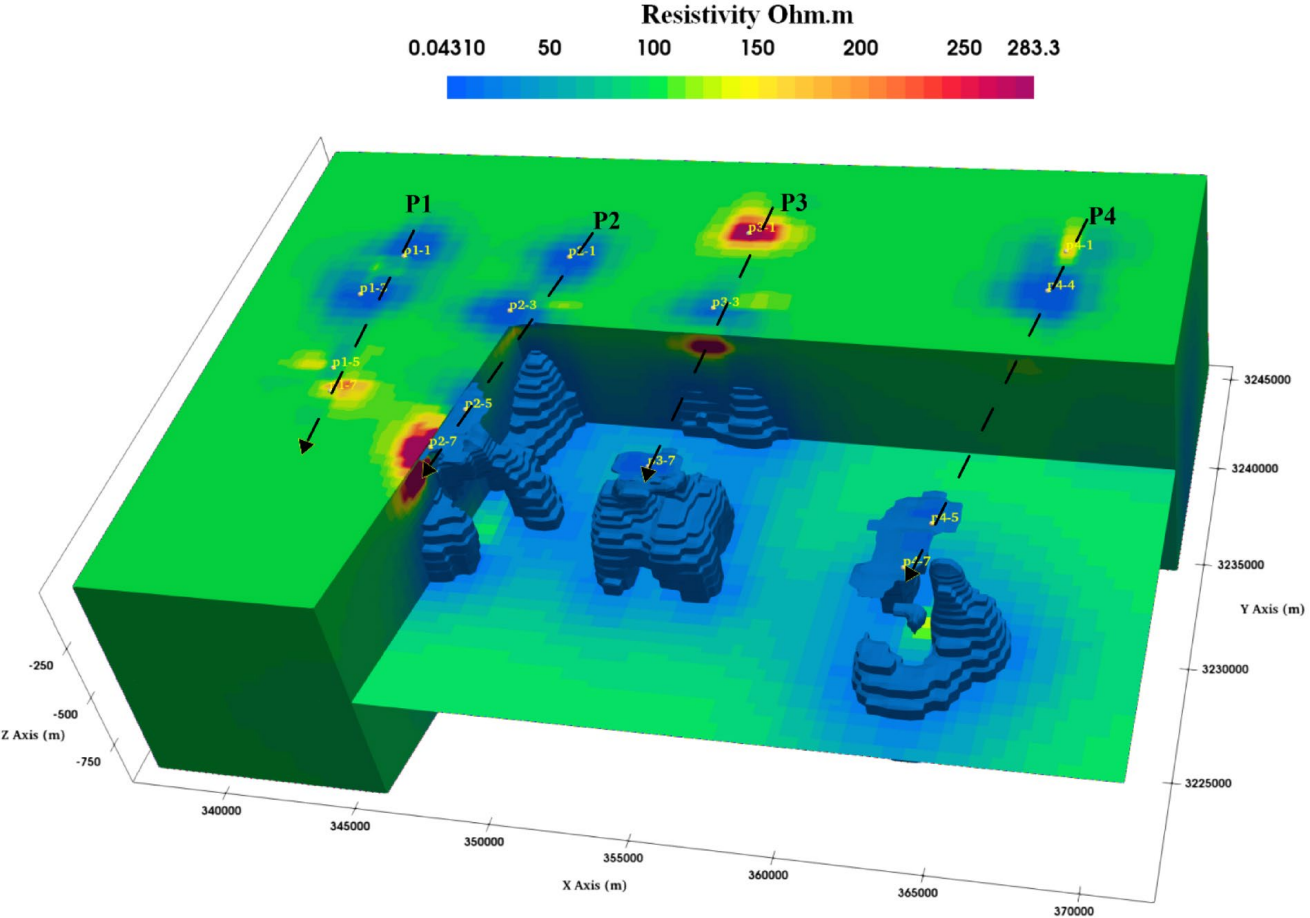
3D resistivity model generated from the inversion of AMT data; the blue iso-surface refers to low resistivity. The 3D model generated by^43^. ParaView (Version 5.11.1). https://www.paraview.org.

The phase tensor skew angle shows high anisotropy and 3D structure at most frequencies, like 2500 Hz, 2.06, and 1.19 Hz. Figure 11a–c present some frequencies, like 80.21 Hz, that show lower values. Presented in Fig. 11b, reflecting low anisotropy. Electric and magnetic time series data were subjected to the Fast Fourier Transform (FFT) to determine the frequencies that dominated the area. The final result of the FFT will be an EDI file that contains the apparent resistivities and phase for each frequency, as shown in Fig. 10, which shows an example of the impedance of profile (p1) as an example of the quality of the data, the quality of data. Most soundings show smoothness on the curve, which means a high quality, unless distorted by the dead band, which describes low signal quality related to the absence of inducing electromagnetic fields at these particular frequencies. Figure 12 shows the 2D cross-section extracted from the 3D model shown in Figs. 13 and 14

The mesh was constructed using the 3D_Grid Software; the inversion was constructed using a cell size of 500 m and padded cells of 7. The model has dimensions of 38 km in the x-direction and 48 km in the y-direction, with 55 cells in the x-direction. In the y-direction, which is 76, the mesh resistivity was 20 Ohm-m before inversion. After iteration 99, the root mean square (RMS) reaches 1.3%.

The 3D inversion resulted using the ModEM algorithm^38,40^ and the plotting of the model and tracing profile p1, p2, p3, and p4 are made using the FFMT MATLAB toolbox^42^ the magnetotelluric stations location are shown in Fig. 8, the distribution of the MT sounding is oriented to cover the north and south banks around the sabkha deposits and the lakes, some areas covered in Sabkha was hard to access, and that is the reason of large AMT station spacing in the west of the study area.

Profiles from p1 to p4, which are shown in Fig. 13, oriented from north to south, were selected to be perpendicular to the seepage zone; all the resistivity cross-sections show changes in resistivity because of different layers in the study area. These variations in resistivity indicate the presence of distinct geological formations, including varying moisture levels and material types. Such insights are crucial for understanding the area’s subsurface conditions and potential groundwater flow. The lower resistivity layers are related to shale and groundwater-saturated zones. The higher resistivity is related to limestone and unsaturated sediments. Still, the general structural view from all the cross-sections is that east–west major faults dissect the area trend that controls the area’s geomorphology, and have the same orientation as the lakes. The seepage may be related to the Miocene aquifer, which occupies the marine deposits of limestone enriched with salt (Moghra formation).

The resistivity-lithology interpretation in this study is not calibrated using borehole data, well logs, or laboratory-derived resistivity readings. Our magnetotelluric models provide robust geoelectrical structures, but the lack of ground-truth resistivity values makes linking resistivity ranges to lithologic units uncertain. Future studies should integrate drill-core data or controlled-source resistivity measurements to improve geological interpretation.

It looks like a structural trend related to recent events controls those trends related to groundwater pathways. Also, the ground morphology may have led to the opening of the groundwater-bearing layers, where the hard, impermeable rocks make a barrier against water, which led to water seepage. The orientation of the seepage zones is nearly east–west; the same direction appears in all the trends estimated from magnetic data. Also, the faults from the magnetotelluric model are oriented in the same direction. We should also refer to the fact that most of the detected structures match what was detected by^1,9,25^

## Conclusion

In this study, we employed geophysical exploration techniques to evaluate the hypothesis that the Siwa Oasis and similar formations may have originated from geological structures. The structural trends inferred from surface and subsurface data indicate a predominant east–west faulting direction, which aligns with the orientation of sabkha deposits and exposed lakes. Surface lineaments identified from the geological map primarily follow northeast-north and east-northeast directions. At the same time, the east–west trend—though less dominant—correlates with the distribution of most lakes in the study area. Shallow trend analysis derived from TDR (tilt derivative) reveals northeast-southwest, northwest-southeast, and east–west orientations. Similarly, the THG (total horizontal gradient) map highlights major structural trends in the northeast-southwest, northwest-southeast, and east–west directions, with the east–west and west-northwest trends being the most prominent. Deeper trend analysis from RTP (reduction-to-the-Pole) magnetic data aligns with the Analytic Signal results, showing dominant northwest-southeast and east–west directions. Depth estimates from 2D magnetic modeling indicate that the basement rock depth in the study area varies between 3400 and 4600 m. 

Additionally, cross-sections from the MT model reveal near-surface east–west faults with resistivity variations attributable to lithological differences and groundwater presence. Our findings suggest groundwater seepage originates from east–west faulting, which creates pathways for groundwater movement or allows saturation along fault planes. This study demonstrates the efficacy of geophysical methods in geological exploration: magnetic data proves invaluable for subsurface structural analysis, while magnetotelluric aids in geological modeling and geoelectric subsurface mapping.

## Supplementary Information

Supplementary Information 1.

## Data Availability

The magnetotelluric and magnetic datasets used and/or analyzed during the current study are available from the corresponding author upon reasonable request. The SRTM Digital elevation model data are available in an online public repository [10.5067/MEaSUREs/SRTM/SRTMGL1N.003].
