# Supplementary figures and images for: Magnetic and magnetotelluric data integration to determine the origin of Siwa Oasis Lakes, Western Desert, Egypt
Source: Sci Rep. 2025 Sep 24;15:32697. doi: 10.1038/s41598-025-20074-x (PMC12460832; doi:10.1038/s41598-025-20074-x)

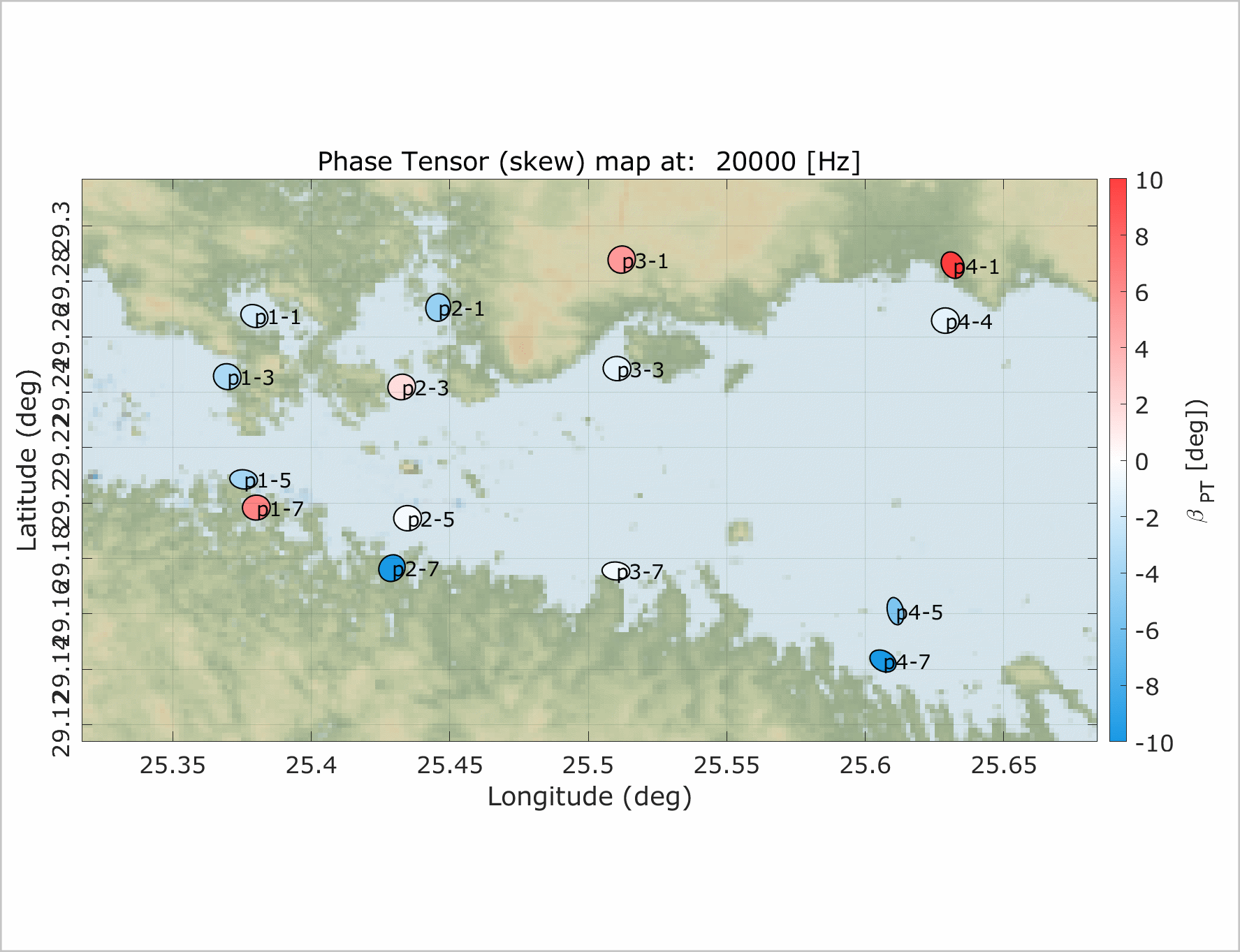

Supplement: Supplementary file 1 — Supplementary Information 1. [file 41598_2025_20074_MOESM1_ESM.gif]
